# Cross-Species, Amplifiable Microsatellite Markers for Neoverrucid Barnacles from Deep-Sea Hydrothermal Vents Developed Using Next-Generation Sequencing

**DOI:** 10.3390/ijms150814364

**Published:** 2014-08-18

**Authors:** Yuichi Nakajima, Chuya Shinzato, Mariia Khalturina, Hiromi Watanabe, Fumio Inagaki, Nori Satoh, Satoshi Mitarai

**Affiliations:** 1 Marine Biophysics Unit, Okinawa Institute of Science and Technology Graduate University, 1919-1 Tancha, Onna, Okinawa 904-0495, Japan; E-Mail: satoshi@oist.jp; 2 Marine Genomics Unit, Okinawa Institute of Science and Technology Graduate University, 1919-1 Tancha, Onna, Okinawa 904-0495, Japan; E-Mails: c.shinzato@oist.jp (C.S.); mariia.khalturina@oist.jp (M.K.); norisky@oist.jp (N.S.); 3 Department of Marine Biodiversity Research, Japan Agency for Marine-Earth Science and Technology, 2-15 Natsushima-cho, Yokosuka, Kanagawa 237-0061, Japan; E-Mail: hwatanabe@jamstec.go.jp; 4 Kochi Institute for Core Sample Research, Japan Agency for Marine-Earth Science and Technology, B-200 Monobe, Nankoku, Kochi 783-8502, Japan; E-Mail: inagaki@jamstec.go.jp

**Keywords:** deep-sea, microsatellites, *Neoverruca*, population genetics

## Abstract

Barnacles of the genus *Neoverruca* are abundant near deep-sea hydrothermal vents of the northwestern Pacific Ocean, and are useful for understanding processes of population formation and maintenance of deep-sea vent faunas. Using next-generation sequencing, we isolated 12 polymorphic microsatellite loci from *Neoverruca* sp., collected in the Okinawa Trough. These microsatellite loci revealed 2–19 alleles per locus. The expected and observed heterozygosities ranged from 0.286 to 1.000 and 0.349 to 0.935, respectively. Cross-species amplification showed that 9 of the 12 loci were successfully amplified for *Neoverruca brachylepadoformis* in the Mariana Trough. A pairwise *F*_ST_ value calculated using nine loci showed significant genetic differentiation between the two species. Consequently, the microsatellite markers we developed will be useful for further population genetic studies to elucidate genetic diversity, differentiation, classification, and evolutionary processes in the genus *Neoverruca*.

## 1. Introduction

Larval dispersal in ecosystems such as deep-sea hydrothermal vents is essential for maintenance of benthic populations and for establishment of new colonies. For vent-endemic benthos, planktonic gametes or larvae provide the only means of migration between hydrothermal vents [1]. However, for most vent-endemic species, migration and dispersal patterns among vent fields have not been well studied. Vent-endemic barnacles belonging to the family Neoverrucidae (Cirripedia: Thoracica) [2] consist of two genera, *Neoverruca* [2] and *Imbricaverruca* [3]. The former is abundant in hydrothermal vent fields of the northwestern Pacific [4,5]. Though the genus *Neoverruca* has no fossil record, the first appearance of close, but extinct ancestors is in the Mesozoic Era [2,6]. Hence, the genus *Neoverruca* is a very primitive barnacle. Presently, the genus *Neoverruca* contains only one described species, *Neoverruca* *brachylepadoformis*. Based on morphology, individuals in the Okinawa Trough and Izu-Ogasawara Arc are distinct from *N. brachylepadoformis* in the Mariana Trough, located south of the Izu-Ogasawara Arc [4,5]. Watanabe *et al.* [5] classified specimens from the Okinawa Trough and Izu-Ogasawara Arc as a new, but unnamed species, designated *Neoverruca* sp., and investigated population structure, focusing on mitochondrial cytochrome oxidase c subunit I (COI). They discovered significant genetic differentiation between the two regions. Further population genetic analysis of this species using high-resolution genetic markers will be needed for detailed estimation of genetic diversity and structure. Therefore, microsatellite markers have been developed for further population genetic studies of *Neoverruca* sp. We isolated and characterized novel polymorphic microsatellite loci for *Neoverruca* sp. using next-generation sequencing and investigated cross-species amplification for *N.* *brachylepadoformis* collected from the Mariana Trough. Furthermore, we estimated the extent of genetic differentiation between *Neoverruca* sp. and *N.* *brachylepadoformis* using developed microsatellite loci, since Watanabe *et al.* [5] previously showed their distinctness using molecular phylogenetic analysis based upon mitochondrial COI.

## 2. Results and Discussion

We obtained 291,118,462 bp (956,987 read pairs) of raw sequence data from genomic DNA of *Neoverruca* sp., and assembled each read pair. We used assembled 736,240 sequences longer than 100 bp (122,338,901 bp total, average 166 bp) for simple sequence repeat identification. Sequence repeats were used for microsatellite detection and primer design. Ninety-six primer pairs were designed for screening amplifiable microsatellite loci (3 mer repeats: 60 loci, 4 mer: 17, 5 mer: 11, 6 mer: 8). Twelve of these loci were successfully amplified and subsequently analyzed in *Neoverruca* sp., although Nsp_11 and Nsp_80 were not amplified in two and one individual(s), respectively. The number of alleles per locus ranged from 2 to 19 (Table 1). Values of observed and expected heterozygosities ranged from 0.286 to 1.000 and 0.349 to 0.935, respectively. Out of 12 loci, two loci, Nsp_09 and Nsp_11, showed significant deviation from Hardy-Weinberg equilibrium, and these two showed the existence of null alleles (99% confidence level). Significant linkage disequilibrium was detected in the combination of Nsp_21-Nsp_37 (*p* < 0.05).

Of the 12 loci isolated and characterized from *Neoverruca* sp., nine were successfully amplified in *N. brachylepadoformis*. Three loci, including Nsp_11 and Nsp_23, which showed high polymorphism in *Neoverruca* sp., were hardly amplified in *N. brachylepadoformis*. The number of individual *N. brachylepadoformis* that showed successful amplification at these nine loci was 14 to 19 (out of 19) (Table 2). These nine loci were also polymorphic in that species, with 3 to 9 alleles per locus (Table 2). Values of observed and expected heterozygosities ranged from 0.063 to 0.786 and 0.174 to 0.855, respectively. In *N. brachylepadoformis*, two loci, Nsp_21 and Nsp_52, showed significant deviation from Hardy-Weinberg equilibrium, but seven other loci did not. A pairwise *F*_ST_ value calculated using nine common loci indicated significant genetic differentiation between the two species (*F*_ST_ = 0.292, *p* = 0.001).

In this study, the number of useful loci and the significance of the pairwise *F*_ST_ implied significant differentiation between *Neoverruca* sp. and *N. brachylepadoformis*. This differentiation is consistent with divergent patterns in molecular phylogenetic trees previously constructed for the COI haplotype [5]. Using nuclear and multiple microsatellite loci, this study robustly demonstrated the genetic differentiation between these two species at the population level.

Further population genetic studies using these markers will enable us to elucidate the level of present and historical dispersal and recruitment between these areas, *i.e.*, between the Okinawa Trough and the Izu-Ogasawara Arc, which influences the formation and maintenance of populations within these species. Furthermore, these markers are expected to help elucidate genetic diversity, differentiation, classification, and evolutionary processes in genus *Neoverruca*. They may even be useful in clarifying the taxonomic diversification concerning this genus.

**Table 1:** Characteristics of the 12 developed polymorphic microsatellite loci in 15 individuals of *Neoverruca* sp. Columns show the locus name, the primer sequence, the repeat motif, the size range of amplification products, including the U19 sequence, numbers of alleles (*N*_A_), observed (*H*_O_) and expected (*H*_E_) heterozygosities, and the index of deviation from Hardy-Weinberg equilibrium (*F*_IS_), and GenBank accession number.

Locus	Repeat Motif	Primer Sequence (5'–3')	Size Range (bp)	*N*_A_	*H*_O_	*H*_E_	*F*_IS_	Accession No.
Nsp_09	(CTTT)3CTTC(TTCC)10TCCCTTCA(TTCC)4	F: AGGAGGCTTTCATGTTTTCC	128–274	11	0.533	0.833	0.360 **	AB971583
		R: U19-AAATGCGTGAGAGTGAAAGG						
Nsp_11 ^††^	(GTGA)12	F: CACTCCTTTCGCGATTCC	298–498	19	0.692	0.935	0.259 *	AB971584
		R: U19-CTACCAGGTGGAGCGTGC						
Nsp_21	(ACACG)11	F: TGAAGCAAGCAATGATAAGC	122–174	8	0.667	0.833	0.200	AB971585
		R: U19-TGTTGCGTCGTGTCG						
Nsp_23	(GCAC)20	F: AACCGGGTTACCCAAAGG	183–373	16	0.867	0.913	0.051	AB971586
		R: U19-TGTGCTGACGGATGTGC						
Nsp_37	(AGA)11	F: U19-CACCCGAGACTTCGATGC	158–167	3	0.467	0.504	0.075	AB971587
		R: TGGGATGAATAAGAGCTGCC						
Nsp_52	(ATC)10	F: CTATACTGGTCGATGCGCC	125–128	2	0.733	0.500	−0.467	AB971588
		R: U19-CCACTTTTGGAGTGCATGG						
Nsp_60	(TCG)10	F: GGATCCGTGTCCCTTATGC	184–202	5	0.400	0.556	0.280	AB971589
		R: U19-TAACTTCAGGGCGCTTCG						
Nsp_68	(GTA)7	F: U19-CTCGTGGGAACCCATCC	93–112	6	1.000	0.758	0.320	AB971590
		R: TCTAAACTCGCGCAAGCC						
Nsp_70	(CAC)7(AAC)2	F: U19-CCTCAGTCTGCACACCC	102–114	5	0.400	0.564	0.291	AB971591
		R: TGGAGGCGATGAAGATGG						
Nsp_73	(AGC)3AAC(AGC)3(AAC)2AGCAAC(AGC)6(AAC)7(AGC)3	F: ATGTGGGTCGGTCTCAGC	122–137	6	0.733	0.704	−0.041	AB971592
		R: U19-TGCATTTGATGTTGCTGC						
Nsp_80 ^†^	(CTA)7	F: U19-TCTGGAACCGGTCTCACC	128–134	3	0.286	0.349	0.182	AB971593
		R: AATAATCCAGAGCGGGACG						
Nsp_81	(ACC)7	F: U19-CGCATAATGACAAACGC	178–187	4	0.467	0.496	0.058	AB971594
		R: CACTGAACATGCAAGCCG						

^†^ Individual(s) that did not show amplification of specific loci (^†^ 1 individual, ^††^ 2 individuals); * Significant deviation from Hardy-Weinberg equilibrium (* *p* < 0.05, ** *p* < 0.01).

**Table 2:** Cross-species amplification for *N. brachylepadoformis* using nine loci developed from *Neoverruca* sp. Columns show the locus name, the number of individuals in which these loci were successfully amplified, the size range of amplification products, including the U19 sequence, numbers of alleles (*N*_A_), observed (*H*_O_) and expected (*H*_E_) heterozygosities, and the index of deviation from Hardy-Weinberg equilibrium (*F*_IS_).

Locus	Succeed	Size Range (bp)	*N*_A_	*H*_O_	*H*_E_	*F*_IS_
Nsp_09	18/19	144–177	7	0.556	0.682	0.186
Nsp_21	16/19	122–137	3	0.063	0.174	0.640 **
Nsp_37	19/19	161–220	9	0.737	0.855	0.138
Nsp_52	16/19	119–134	5	0.438	0.604	0.275 ***
Nsp_60	18/19	160–196	4	0.278	0.335	0.171
Nsp_68	14/19	93–118	8	0.786	0.747	−0.051
Nsp_70	18/19	108–117	4	0.667	0.500	−0.333
Nsp_73	18/19	110–125	6	0.611	0.645	0.053
Nsp_81	19/19	178–187	3	0.316	0.476	0.337

* Significant deviation from Hardy-Weinberg equilibrium (** *p* < 0.01, *** *p* < 0.001).

## 3. Experimental Section

For isolation of whole genomic DNA, *Neoverruca* sp. was collected at the Iheya Depression (27°32.994'N/126°58.158'E, 1402 m depth) in the Okinawa Trough during cruise of R/V “Kaiyo” (Cruise No. KY11-02 Leg. 2), using the remotely operated vehicle (ROV) “Hyper-Dolphin” (Dive No. HPD#1245). Specimens were preserved in ethanol, and genomic DNA was isolated using proteinase K and phenol-chloroform extraction. DNA was further purified using ethanol precipitation and a QIAquick PCR purification kit (Qiagen, Hilden, Germany). Extracted DNA was sequenced as 300 bp paired end reads using a MiSeq sequencer (Illumina, San Diego, CA, USA), according to manufacturer’s instructions. Sequencing adapters were trimmed with fastq-mcf in ea-utils version 1.1.2-537 [7] and sequences of all read pairs were assembled using fastq-join in ea-utils [7]. Then assembled sequences longer than 100 bp were selected. Detection of simple sequence repeats and PCR primer design in each assembled sequence were performed with PAL_FINDER version 0.02.04 [8]. Then we removed redundancies in the assembled sequences in which PAL_FINDER detected simple sequence repeats and designed primers using CDHIT-EST [9]. In order to select microsatellite loci that may be highly variable, we selected primer pairs amplifying longer repeat stretches (thresholds: 3 mer is 5 repeats or more, 4 to 6 mers are 10 or more, respectively).

**Figure 1:**
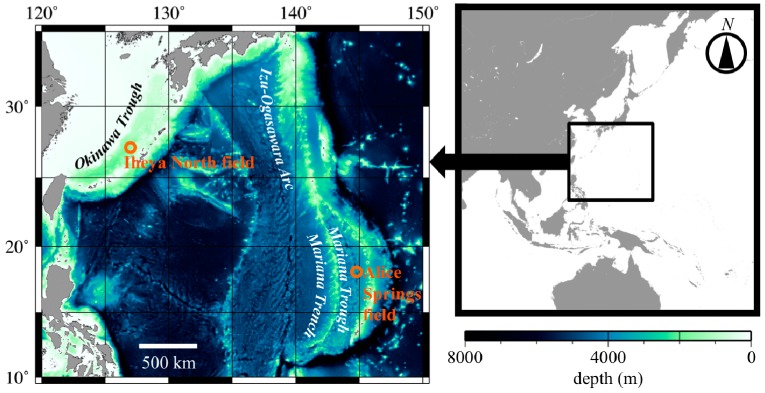
Sampling sites for *Neoverruca*. Iheya North field (for *Neoverruca* sp.) is located in the Okinawa Trough and the Alice Springs field (*N. brachylepadoformis*) is in the Mariana Trough.

To characterize microsatellite loci, we screened 15 individuals of *Neoverruca* sp. collected at the Iheya North field (27°47.196'N/126°53.862'E, 990 m depth and 27°47.226'N/126°53.832'E, 977 m depth, Figure 1) in the Okinawa Trough during cruise of the R/V “Natsushima” (Cruise No. NT00-08), using the submersible, “Shinkai 2000” (Dive No. 2K#1192 and 2K#1194). Specimens collected were preserved in ethanol, and genomic DNA was extracted using a DNeasy Blood & Tissue Kit (Qiagen). To assess amplification and evaluate polymorphism of the designed primer sets, we performed PCR as follows. The reaction mixture (5 µL) contained <20 ng of template genomic DNA, AmpliTaq Gold 360 Master Mix (Applied Biosystems, Foster City, CA, USA), and three primers for each locus: a non-tailed forward primer (0.5 µM), a reverse primer with a U19 (5'-GGTTTTCCCAGTCACGACG-3') tail (0.5 µM), and a U19 primer (0.5 µM) fluorescently labeled with FAM, VIC, NED, or PET, based on the method of Schuelke [10]. PCR amplification was performed under the following conditions: 95 °C for 5 min; followed by 35 cycles at 95 °C for 30 s, 54 °C for 30 s, and 72 °C for 1 min, with a final extension at 72 °C for 5 min. Amplified PCR products were analyzed using an automated capillary-based DNA sequencer (ABI 3130xl Genetic Analyzer, Applied Biosystems) and GeneMapper version 3.7 (Applied Biosystems). For successfully amplified microsatellite loci for *Neoverruca* sp., cross-species amplification was further examined in *N. brachylepadoformis* (19 individuals) with aforementioned procedures and conditions. These specimens were collected at the Alice Springs field (18°12.599'N/144°42.431'E, 3640 m depth, Figure 1) in the Mariana Trough during a cruise of the R/V “Atlantis II” using the submersible “Alvin”.

For successfully amplified microsatellite loci, the number of alleles, expected and observed heterozygosity, and deviation index from Hardy-Weinberg equilibrium (*F*_IS_) were calculated with GenAlEx version 6.5 [11]. The software Micro-Checker version 2.2.3 [12] performed 10,000 randomizations to check for null alleles at each locus at the 95% and 99% confidence levels. For all successful loci, linkage disequilibrium was estimated with Genepop version 4.2 [13,14,15] under the following Markov chain parameters: 10,000 dememorizations, 1000 batches, 10,000 iterations per batch. For evaluation of outcomes in cross-species amplification, the number of successful microsatellite loci was counted in *N. brachylepadoformis*. In addition, the number of alleles, expected and observed heterozygosity, and deviation index from Hardy-Weinberg equilibrium were also calculated in *N. brachylepadoformis*. To estimate the level of genetic differentiation between populations of *Neoverruca* sp. and *N. brachylepadoformis*, pairwise *F*_ST_ was calculated using GenAlEx focusing cross-species amplifiable loci. The test for statistical significance was based on 999 random permutations.

## 4. Conclusions

Twelve microsatellite loci developed in this study were successfully amplified and should have utility for further population genetics of *Neoverruca* sp. to estimate the processes of population formation and maintenance. In addition, nine of the primers can amplify microsatellites in *N. brachylepadoformis*. These markers are expected to help elucidate genetic diversity, differentiation, classification, and evolutionary processes in the genus *Neoverruca*.
